# Supplementary material for: A UMLS-based spell checker for natural language processing in vaccine safety
Source: BMC Med Inform Decis Mak. 2007 Feb 12;7:3. doi: 10.1186/1472-6947-7-3 (PMC1805499; doi:10.1186/1472-6947-7-3)
Supplement: Additional file 13 — RAP application directory. Sets up directory for RAP (RDF application for PHP) source code [file 1472-6947-7-3-S13.gz › rap/doc/phpdoc/infModel/InfModelB.html]

xml version="1.0" encoding="iso-8859-1"?


Docs For Class InfModelB


## Class InfModelB

Description

Description |
Methods (details)

A InfModelB extends the InfModel Class, with a backward chaining algorithm.

Only the loaded or added base-triples are stored. A find-query evaluates the inference rules and recursively tries to find the statements. InfModelB memorises "Dead-Ends" until the next add() command, thus makin a second find much faster. InfModelB is safe for loops in Ontologies, that would cause infinite loops. WARNING: A find(null,null,null) might take very long.

History:

- 10-07-2004 : Function findFirstMatchingStatement() added paramter $offset
  to set an search offset
- 09-10-2004 : First version of this class.
- 09-15-2004 : Added Index over InRule Entailments
  to increase performance

- access: public
- author: Daniel Westphal <at d-westphal dot de mail at d-westphal dot de>
- version: V0.9.1

Located in /infModel/InfModelB.php (line 30)

```
Object
   |
   --Model
      |
      --MemModel
         |
         --InfModel
            |
            --InfModelB
```

Method Summary

Description |
Methods (details)

InfModelB
InfModelB
([string $baseURI = null])

void
add
(object Statement $statement)

boolean
contains
(object Statement &$statement)

object MemModel
find
(object Node $subject, object Node $predicate, object Node $object)

object FindIterator
&findAsIterator
([mixed $sub = null], [mixed $pred = null], [mixed $obj = null])

object Statement
findFirstMatchingStatement
(object Node $subject, object Node $predicate, object Node $object, integer $offset)

object MemModel
&getBaseMemModel
()

object MemModel
&getMemModel
()

object StatementIterator
&getStatementIterator
()

boolean
remove
(object Statement $statement)

boolean
saveAs
(string $filename, [string $type = 'rdf'])

integer
size
(boolean 0)

string
toString
()

string
toStringIncludingTriples
()

void
writeAsHtml
()

void
writeAsHtmlTable
()

string
writeRdfToString
()

Methods

Description |
Methods (details)

Constructor InfModelB (line 49)

Constructor You can supply a base\_uri

- access: public

InfModelB

InfModelB
([string $baseURI = null])

- string
  $baseURI

add (line 64)

Adds a new triple to the Model without checking, if the statement is already in the Model. So if you want a duplicate free Model use the addWithoutDuplicates() function (which is slower then add())

- throws: PhpError
- access: public

void

add
(object Statement $statement)

- object Statement
  $statement

---

Redefinition of:

InfModel::add()
:   Adds a new triple to the Model without checking if the statement is already in the Model.

contains (line 236)

Tests if the Model contains the given triple.

TRUE if the triple belongs to the Model; FALSE otherwise.

- access: public

boolean

contains
(object Statement &$statement)

- object Statement
  &$statement

---

Redefinition of:

MemModel::contains()
:   Tests if the MemModel contains the given triple.

find (line 90)

General method to search for triples.

NULL input for any parameter will match anything. Example: $result = $m->find( NULL, NULL, $node ); Finds all triples with $node as object. Returns an empty MemModel if nothing is found. To improve the search speed with big Models, call index(INDEX\_TYPE) before seaching.

It recursively searches in the statements and rules to find matching statements

- throws: PhpError
- access: public

object MemModel

find
(object Node $subject, object Node $predicate, object Node $object)

- object Node
  $subject
- object Node
  $predicate
- object Node
  $object

---

Redefinition of:

MemModel::find()
:   General method to search for triples.

findAsIterator (line 511)

Returns a FindIterator for traversing the MemModel.

Disabled in InfModelB

- access: public

object FindIterator

&findAsIterator
([mixed $sub = null], [mixed $pred = null], [mixed $obj = null])

---

Redefinition of:

MemModel::findAsIterator()
:   Returns a FindIterator for traversing the MemModel.

findFirstMatchingStatement (line 270)

Searches for triples and returns the first matching statement.

NULL input for any parameter will match anything. Example: $result = $m->findFirstMatchingStatement( NULL, NULL, $node ); Returns the first statement of the MemModel where the object equals $node. Returns an NULL if nothing is found. You can define an offset to search for. Default = 0

- access: public

object Statement

findFirstMatchingStatement
(object Node $subject, object Node $predicate, object Node $object, integer $offset)

- object Node
  $subject
- object Node
  $predicate
- object Node
  $object
- integer
  $offset

---

Redefinition of:

MemModel::findFirstMatchingStatement()
:   Searches for triples and returns the first matching statement.

getBaseMemModel (line 340)

Create a MemModel containing only the base triples (without inferred statements) of the current InfModelB

- access: public

object MemModel

&getBaseMemModel
()

getMemModel (line 324)

Create a MemModel containing all the triples (including inferred statements) of the current InfModelB

- access: public

object MemModel

&getMemModel
()

getStatementIterator (line 290)

Returns a StatementIterator for traversing the Model.

- access: public

object StatementIterator

&getStatementIterator
()

---

Redefinition of:

MemModel::getStatementIterator()
:   Returns a StatementIterator for traversing the MemModel.

remove (line 461)

Removes the triple from the MemModel.

TRUE if the triple is removed. FALSE otherwise.

Checks, if it touches any statements, that added inference rules to the model

- throws: PhpError
- access: public

boolean

remove
(object Statement $statement)

- object Statement
  $statement

---

Redefinition of:

MemModel::remove()
:   Removes the triple from the MemModel.

saveAs (line 394)

Saves the RDF,N3 or N-Triple serialization of the full InfModelB (including inferred triples) to a file.

You can decide to which format the model should be serialized by using a corresponding suffix-string as $type parameter. If no $type parameter is placed this method will serialize the model to XML/RDF format. Returns FALSE if the InfModelB couldn't be saved to the file.

- access: public

boolean

saveAs
(string $filename, [string $type = 'rdf'])

- string
  $filename
- string
  $type

---

Redefinition of:

MemModel::saveAs()
:   Saves the RDF,N3 or N-Triple serialization of the MemModel to a file.

size (line 308)

Number of all inferable triples in the Model.

WARNING: uses a find(null,null,null) to find all statements! (might take a while)

- access: public

integer

size
(boolean 0)

- boolean
  0

---

Redefinition of:

MemModel::size()
:   Number of triples in the MemModel

toString (line 357)

Short Dump of the InfModelB.

- access: public

string

toString
()

---

Redefinition of:

MemModel::toString()
:   Short Dump of the MemModel.

toStringIncludingTriples (line 368)

Dumps of the InfModelB including ALL inferable triples.

- access: public

string

toStringIncludingTriples
()

---

Redefinition of:

MemModel::toStringIncludingTriples()
:   Dumps of the MemModel including all triples.

writeAsHtml (line 407)

Writes the RDF serialization of the Model including ALL inferable triples as HTML.

- access: public

void

writeAsHtml
()

---

Redefinition of:

MemModel::writeAsHtml()
:   Writes the RDF serialization of the MemModel as HTML.

writeAsHtmlTable (line 424)

Writes the RDF serialization of the Model including ALL inferable triples as HTML table.

- access: public

void

writeAsHtmlTable
()

---

Redefinition of:

MemModel::writeAsHtmlTable()
:   Writes the RDF serialization of the MemModel as HTML table.

writeRdfToString (line 439)

Writes the RDF serialization of the Model including ALL inferable triples.

- access: public

string

writeRdfToString
()

---

Redefinition of:

MemModel::writeRdfToString()
:   Writes the RDF serialization of the MemModel as HTML table.

#### Inherited Methods


Inherited From InfModel

> InfModel::InfModel()  
> InfModel::add()  
> InfModel::findRegex()  
> InfModel::findVocabulary()  
> InfModel::getSchema()  
> InfModel::replace()

Inherited From MemModel

> MemModel::MemModel()  
> MemModel::add()  
> MemModel::addModel()  
> MemModel::addNamespace()  
> MemModel::addParsedNamespaces()  
> MemModel::addWithoutDuplicates()  
> MemModel::close()  
> MemModel::contains()  
> MemModel::containsAll()  
> MemModel::containsAny()  
> MemModel::equals()  
> MemModel::find()  
> MemModel::findAsIterator()  
> MemModel::findCount()  
> MemModel::findFirstMatchingStatement()  
> MemModel::findRegex()  
> MemModel::findVocabulary()  
> MemModel::getIndexType()  
> MemModel::getParsedNamespaces()  
> MemModel::getStatementIterator()  
> MemModel::index()  
> MemModel::intersect()  
> MemModel::isEmpty()  
> MemModel::isIndexed()  
> MemModel::iterFind()  
> MemModel::rdqlQuery()  
> MemModel::rdqlQueryAsIterator()  
> MemModel::reify()  
> MemModel::remove()  
> MemModel::removeNamespace()  
> MemModel::replace()  
> MemModel::saveAs()  
> MemModel::setBaseURI()  
> MemModel::size()  
> MemModel::subtract()  
> MemModel::toString()  
> MemModel::toStringIncludingTriples()  
> MemModel::unite()  
> MemModel::writeAsHtml()  
> MemModel::writeAsHtmlTable()  
> MemModel::writeRdfToString()

Inherited From Model

> Model::Model()  
> Model::findForward()  
> Model::getBaseURI()  
> Model::getMemModelByRDQL()  
> Model::getOntModel()  
> Model::getResmodel()  
> Model::load()  
> Model::visualize()

Inherited From Object

> Object::toString()

Documentation generated on Thu, 7 Jul 2005 13:41:38 +0200 by phpDocumentor 1.3.0RC3
